# Efficacy and safety of arimoclomol in Niemann‐Pick disease type C: Results from a double‐blind, randomised, placebo‐controlled, multinational phase 2/3 trial of a novel treatment

**DOI:** 10.1002/jimd.12428

**Published:** 2021-09-07

**Authors:** Eugen Mengel, Marc C. Patterson, Rosalia M. Da Riol, Mireia Del Toro, Federica Deodato, Matthias Gautschi, Stephanie Grunewald, Sabine Grønborg, Paul Harmatz, Bénédicte Héron, Esther M. Maier, Agathe Roubertie, Saikat Santra, Anna Tylki‐Szymanska, Simon Day, Anne Katrine Andreasen, Marie Aavang Geist, Nikolaj Havnsøe Torp Petersen, Linda Ingemann, Thomas Hansen, Thomas Blaettler, Thomas Kirkegaard, Christine í Dali

**Affiliations:** ^1^ SphinCS GmbH Institute of Clinical Science for LSD Hochheim Germany; ^2^ Departments of Neurology, Pediatrics and Medical Genetics Mayo Clinic Rochester Minnesota USA; ^3^ Regional Coordination Center for Rare Diseases Academic Hospital ‘Santa Maria della Misericordia’ Udine Italy; ^4^ Pediatric Neurology Department Vall d'Hebron University Hospital Barcelona Spain; ^5^ Division of Metabolism Ospedale Pediatrico Bambino Gesù, IRCCS Rome Italy; ^6^ Department of Paediatrics, Division of Endocrinology, Diabetology and Metabolism, and Institute of Clinical Chemistry, Inselspital University Hospital Bern, University of Bern Bern Switzerland; ^7^ Department of Metabolic Medicine Great Ormond Street Hospital, Institute of Child Health, UCL, NIHR Biomedical Research Center London UK; ^8^ Centre for Inherited Metabolic Diseases Copenhagen University Hospital (Rigshospitalet) Copenhagen Denmark; ^9^ Gastroenterology and Hepatology UCSF Benioff Children's Hospital Oakland Oakland California USA; ^10^ Department of Pediatric Neurology, Reference Centre for Lysosomal Diseases University Hospital Armand Trousseau Paris France; ^11^ Department of Inborn Errors of Metabolism University of Munich Children's Hospital Munich Germany; ^12^ Department of Neuropediatrics Centre Hospitalier Universitaire de Montpellier Montpellier France; ^13^ Department of Inherited Metabolic Disorders Birmingham Children's Hospital Birmingham UK; ^14^ Department of Paediatrics, Nutrition and Metabolic Diseases The Children's Memorial Institute Warsaw Poland; ^15^ Biostatistics Clinical Trials Consulting & Training Limited Buckingham UK; ^16^ Orphazyme A/S Copenhagen Denmark

**Keywords:** arimoclomol, biomarker, double‐blindplacebo‐controlled, heat shock protein, Niemann‐Pick disease type C, NPC clinical severity scale

## Abstract

Niemann‐Pick disease type C (NPC) is a rare, genetic, progressive neurodegenerative disorder with high unmet medical need. We investigated the safety and efficacy of arimoclomol, which amplifies the heat shock response to target NPC protein misfolding and improve lysosomal function, in patients with NPC. In a 12‐month, prospective, randomised, double‐blind, placebo‐controlled, phase 2/3 trial (ClinicalTrials.gov identifier: NCT02612129), patients (2‐18 years) were randomised 2:1 to arimoclomol:placebo, stratified by miglustat use. Routine clinical care was maintained. Arimoclomol was administered orally three times daily. The primary endpoint was change in 5‐domain NPC Clinical Severity Scale (NPCCSS) score from baseline to 12 months. Fifty patients enrolled; 42 completed. At month 12, the mean progression from baseline in the 5‐domain NPCCSS was 0.76 with arimoclomol vs 2.15 with placebo. A statistically significant treatment difference in favour of arimoclomol of −1.40 (95% confidence interval: −2.76, −0.03; *P* = .046) was observed, corresponding to a 65% reduction in annual disease progression. In the prespecified subgroup of patients receiving miglustat as routine care, arimoclomol resulted in stabilisation of disease severity over 12 months with a treatment difference of −2.06 in favour of arimoclomol (*P* = .006). Adverse events occurred in 30/34 patients (88.2%) receiving arimoclomol and 12/16 (75.0%) receiving placebo. Fewer patients had serious adverse events with arimoclomol (5/34, 14.7%) vs placebo (5/16, 31.3%). Treatment‐related serious adverse events (n = 2) included urticaria and angioedema. Arimoclomol provided a significant and clinically meaningful treatment effect in NPC and was well tolerated.

## 1 INTRODUCTION

Niemann‐Pick disease type C (NPC) is a rare, progressive neurodegenerative disease.^1^, ^2^ As a result of dysfunctional NPC proteins, lysosomal function is impaired and multiple lipid species accumulate.^3^, ^4^ Recent studies show that NPC1 and NPC2 proteins are involved in cholesterol efflux from late lysosomal and endosomal compartments and regulate cholesterol content within membranes.^5^, ^6^ Knowledge on the full spectrum of functions of the NPC1/NPC2 proteins is not complete but continues to expand through detailed structural studies.^5^, ^6^

The clinical presentation and progression of NPC is heterogeneous, depends on age at the time of neurological symptom onset, and includes loss of motor function, swallowing, and speech, as well as cognitive impairment.^2^, ^7^, ^8^, ^9^ Individuals with infantile onset of neurological symptoms generally have a more aggressive disease course than patients with juvenile or late‐onset disease.^2^ There is no cure for NPC. However, in Europe, miglustat is an approved treatment that has demonstrated a modest effect on slowing disease progression in pivotal trials^10^, ^11^, ^12^; long‐term follow‐up suggests treatment benefit.^13^, ^14^ Miglustat is not approved for NPC in the USA but is often used off‐label.

Most mutations in NPC are missense mutations (70%‐80%), resulting in misfolding and premature degradation of NPC1 protein.^15^, ^16^, ^17^ Less common (20%‐30%) are splicing, frameshift, or premature stop mutations (collectively designated functional null mutations) causing truncated and deficient NPC1 protein,^15^, ^16^, ^17^ predictive of particularly severe disease progression and early onset when present on both alleles.^15^, ^18^ With the exception of these double functional null mutations, a consistent genotype/phenotype relationship in NPC is difficult to establish.^4^

During cellular stress, the heat shock response (HSR) is part of the natural cellular defence which prevents protein misfolding and promotes lysosomal homeostasis.^19^, ^20^ Activation of the HSR leads to production of molecular chaperones, the heat shock proteins (HSPs), and especially HSP70, which is critical in the correct processing and folding of the NPC1 protein.^21^, ^22^ The HSR is linked directly to lysosomal function and integrity, in part through HSP70‐mediated augmentation of sphingolipid‐degrading enzymes, stabilisation of lysosomal membranes, and protection from cell death.^20^, ^21^, ^23^, ^24^

Arimoclomol is an orally available small molecule that crosses the blood‐brain barrier, as evidenced by its presence in cerebrospinal fluid of treated patients with amyotrophic lateral sclerosis.^25^ Arimoclomol amplifies the natural response to cellular stress by inducing the HSR and production of HSPs to prevent protein misfolding, but also by more direct facilitation of lysosomal function. Thus, arimoclomol preserves cellular function and prevents cell death in cells experiencing lysosomal stress.^20^, ^21^, ^26^, ^27^, ^28^, ^29^

We conducted the present trial (ClinicalTrials.gov identifier: NCT02612129) to evaluate the safety and efficacy of arimoclomol in patients with NPC. The primary endpoint was the 5‐domain NPC Clinical Severity Scale (NPCCSS) score, as described by Mengel et al.^1^ and Patterson et al.^30^

## 2 METHODS

### 2.1 Study design

**FIGURE 1:**
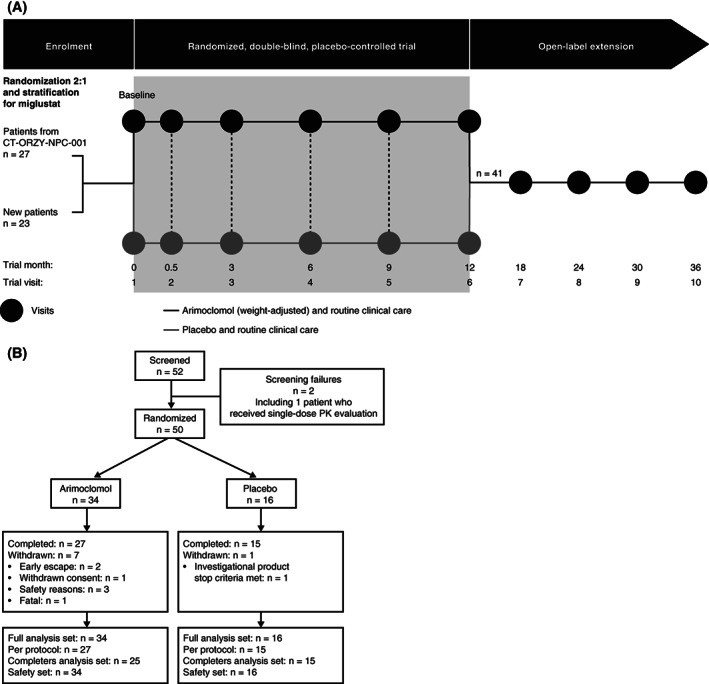
A, CT‐ORZY‐NPC‐002 trial design. B, Patient flow. Completers analysis set excluded two patients in the arimoclomol group who did not have assessments at 12 months. PK, pharmacokinetic

This was a 12‐month, prospective, randomised, double‐blind, placebo‐controlled, phase 2/3 multinational trial in which all patients continued to receive routine clinical care (Figure 1A). The trial was performed at 14 clinical sites in 9 countries (Denmark, France, Germany, Italy, Poland, Spain, Switzerland, UK, and USA).

The trial adhered to the Declaration of Helsinki, International Conference on Harmonisation and Good Clinical Practice standards, and applicable local guidelines.^31^, ^32^ The trial protocol and associated documentation were approved by the relevant independent ethics committees and/or institutional review boards.

### 2.2 Participants

Eligible patients were male or female patients aged 2 to 18 years who fulfilled the following criteria: genetically confirmed mutations in both alleles of *NPC1* or *NPC2*, or a mutation in only one allele of *NPC1* or *NPC2* plus either positive filipin staining or elevated cholestane‐triol level (>2 × upper limit of normal); at least one neurological sign of disease; ability to walk independently or with assistance; and if treated with miglustat, being on stable dosing for at least 6 months. Patients who completed our NPC observational study^1^ end‐of‐study visit could enrol directly into this trial (N = 27). Exclusion criteria included: concurrent participation in other trials (non‐interventional registry participation was allowed); severe liver or renal insufficiency; receiving treatment with any investigational medicinal product within 4 weeks of trial enrolment or during the trial; being neurologically asymptomatic or having severe, uncontrolled epileptic seizures (see Supplementary Materials); and failure to provide written informed consent. Written informed consent was obtained at trial initiation from either the patient or their legal guardian.

### 2.3 Randomisation and masking

Patients enrolled in the trial were stratified by use of miglustat at baseline. An interactive response technology system assigned treatment to each patient via a randomisation list. Patients in both strata were randomised 2:1 to receive arimoclomol or placebo. Given the ultra‐rare condition, and expected rate of progression, sample size and trial duration were primarily informed by feasibility and not by a formal sample size calculation. Up to 52 patients with NPC were planned to be randomised, to have at least 40 evaluable patients' data at the 12 months landmark visit.

To ensure blinding, all investigational products and their packaging (arimoclomol 16 mg, 31 mg, and 62 mg capsules, and placebo capsules) were indistinguishable in terms of composition, texture, appearance, solubility, smell, and flavour.

### 2.4 Procedures

The screening visit (visit 1) included a baseline assessment, randomisation assignment, and pharmacokinetic (PK) assessment (in all patients aged <12 years, regardless of treatment allocation; see Supplementary Materials). The first dose of arimoclomol or placebo was given within 1 week of randomisation. Safety assessments were performed at visit 2 (7‐14 days after start of treatment) and then every 3 months during the blinded period (visits 3‐6). Monthly telephone follow‐ups were performed to evaluate safety, confirm patients' weights, and assess treatment compliance (Figure 1A).

Routine clinical care was maintained throughout the trial (including administration of miglustat). Each patient was randomised to receive arimoclomol or placebo three times daily. Arimoclomol was administered orally or by feeding tube at 93 to 372 mg/day based on the patient's body weight as described in Table S4. Arimoclomol was manufactured by Juniper Pharma (Nottingham, UK).

During the 12‐month treatment phase, efficacy assessments for the primary endpoint, the 5‐domain NPCCSS scores, and for the non‐disease specific Clinical Global Impression—Improvement scale (CGI‐I) scores were performed at baseline and after 3, 6, 9, and 12 months of treatment; all other efficacy and biomarker assessments were performed at baseline and after 6 and 12 months of treatment.

An independent Data Safety Monitoring Board (DSMB), consisting of three experts who had access to unblinded data, reviewed safety data four times during the blinded phase. For patients in either treatment group with rapid disease progression, an “early escape clause” could be invoked if a particular set of criteria were met (assessed by the investigator and approved by the sponsor). In these events, blinded study drug dosing was suspended and “rescue” arimoclomol treatment was initiated while patients continued with their current protocol schedule (Figure 1B).

### 2.5 Outcomes

#### 2.5.1 Primary endpoint

The primary endpoint was change from baseline in NPC severity at 12 months as assessed by the 5‐domain NPCCSS, an abbreviated assessment tool originating from the 17‐domain NPCCSS developed by Yanjanin et al.^33^ The fully validated 5‐domain NPCCSS^1^, ^30^ comprises the domains determined to be most clinically relevant to patients, caregivers, and clinicians: ambulation, cognition, fine motor skills, speech, and swallowing.^34^ The total aggregated 5‐domain NPCCSS score ranges from 0 to 25, with a higher score indicating more severe clinical impairment.

#### 2.5.2 Subgroup analyses of primary endpoint

NPC is a heterogeneous disease, and patients aged 2 to 18 years present with a large spectrum of disease manifestations.^2^, ^35^ The group of children <4 years old includes patients with mild manifestations and patients with aggressive, early fatal disease. To account for expected heterogeneity, a subgroup analysis of patients ≥4 years of age was predefined.^2^, ^9^

In the EU, miglustat is indicated for the treatment of progressive neurological manifestations in patients with NPC. However, not all patients are candidates for miglustat treatment (eg, patients without neurologic involvement and patients with advanced neurological disease), and it is recommended that the benefit of treatment should be evaluated on a regular basis (eg, every 6 months).^35^, ^36^ An analysis of the subpopulation of patients receiving miglustat was prespecified to elucidate the effect of arimoclomol in patients on background miglustat treatment. Overall, the subgroups of patients ≥4 years of age, and patients on miglustat treatment, were expected to be more homogeneous with respect to baseline demographics and disease characteristics.

Two *post hoc* subgroup analyses were also conducted. In the first *post hoc* analysis, patients with double functional null mutations in *NPC1* were excluded. The presence of functional null mutations on both alleles of NPC1 (double functional null) is rare in patients with NPC, and is strongly predictive of early onset and rapid disease progression.^15^, ^37^ Patients with double functional null mutations do not produce any full length NPC1 protein, but these patients could still benefit from arimoclomol treatment via its direct HSP70‐mediated stabilisation of lysosomal membranes independent of NPC proteins.^21^, ^22^ As heterogeneity in the mutation types could have an impact on the efficacy signal, this additional analysis limited to patients with more similar genotypes was conducted to assess whether this indeed would result in enhancement of the efficacy signal. In the second *post hoc* analysis, only patients with an annual severity increment score (ASIS) of 0.5 to 2.0 were included. Cortina‐Borja et al. suggested that by applying differential ASIS cut‐off points of 0.5 to 2.0, which excludes the very mild and very severe patients, trial cohorts may be stratified to obtain a more homogeneous patient population that, if untreated, would change in clinical score within the typical period of a clinical trial.^34^

#### 2.5.3 Secondary endpoints

A key secondary endpoint was responder analysis of CGI‐I scores (responder defined as stable or improved) at 12 months compared with baseline; this was implemented as a co‐primary endpoint for the FDA. Other key secondary endpoints were: responder analysis of 5‐domain NPCCSS scores (defined as stable or improved) at month 12 vs baseline; time to worsening on 5‐domain NPCCSS (defined as the time until the patient worsened by 2 points vs baseline); proportion of patients worsening on 5‐domain NPCCSS at 6 and 12 months by 2 points on the 5‐domain NPCCSS; and change in 17‐domain NPCCSS (excluding hearing domains) at 12 months. Additional secondary endpoints included: change from baseline in Scale for Assessment and Rating of Ataxia (SARA) score at 6 and 12 months; change in the nine‐hole peg test (9‐HPT) result at 6 and 12 months; change in health‐related quality of life (HRQoL) as measured by the 5‐dimension 3‐level EuroQol questionnaire, youth version (EQ‐5D‐3L Y) proxy at 6 and 12 months; change in individual 5‐domain NPCCSS scores at month 12; and NPC clinical database (NPC‐cdb) score changes from baseline at trial time points. The NPC‐cdb score aims to reflect clinical status; an increase in score indicates a reduction in the patient's abilities. The score was calculated as defined by Stampfer et al.^8^

#### 2.5.4 Biomarkers

HSP70 was assessed as a marker of pharmacodynamic activity of arimoclomol. Cholestane‐triol in serum and unesterified cholesterol in peripheral blood mononuclear cells (PBMCs) are measures of lipid burden in NPC. Cholestane‐triol in serum, unesterified cholesterol, and HSP70 were investigated as previously reported.^1^ After completion of the trial, we also investigated Lyso‐SM‐509, which is related to disease pathology^38^ and correlates with disease severity and NPC‐specific biomarkers^39^, ^40^ (see Supplementary Materials for further details).

#### 2.5.5 Safety

All treatment‐emergent adverse events (TEAEs) and serious TEAEs were recorded, as were any changes in results of physical examinations, vital signs, electrocardiographic results, and standard haematology and clinical chemistry findings. All events were classified using the Medical Dictionary for Regulatory Activities version 19.0. Arimoclomol inhibits the organic cation transporter 2 involved in creatinine secretion in the kidneys leading to a reversible increase in serum creatinine and/or decrease in mean creatinine clearance. Therefore, to avoid possible treatment effects on creatinine levels leading to unblinding of the investigators, visit 2 (7‐14 days after start of treatment) creatinine test results were reviewed by an independent expert only.

### 2.6 Statistical analysis

No formal statistical calculation was used to determine sample size. Efficacy endpoints were analysed in the full analysis set, which included all patients who were randomised and started study treatment (excluding the PK analysis single dose of arimoclomol [see Supplementary Materials]). All dosed patients were included in the analysis of efficacy; patients were included until and including the assessment at which rescue medication was initiated. The safety set comprised the same patients as the full analysis set.

Data for continuous outcomes including the primary endpoint were analysed under an efficacy (de jure) estimand with a mixed model for repeated measures (MMRM), with main effects factors for treatment, visit, miglustat stratum, and the corresponding baseline value and the interaction term between visit and treatment. Baseline disease severity scores were chosen as a covariate in the primary analysis to account for anticipated differences between individuals and treatment arms with regards to severity and to allow the analysis of change from baseline to mirror that of absolute values. Missing data were not imputed for the primary endpoint analysis but were imputed for sensitivity analyses. Sensitivity analyses were performed both under the primary estimand and under a *de facto* “treatment policy” estimand. The primary MMRM model was applied on the prespecified subgroup levels with enough patients to substantiate a formal analysis. See Supplementary Materials for additional analyses. All statistical analyses were performed using SAS software version 9.3 (SAS Institute Inc., Cary, North Carolina). All hypothesis testing was carried out using a 5% (two‐sided) significance level.

## 3 RESULTS

### 3.1 Participants

The trial was conducted between June 2016 and June 2018; 52 patients were screened, and 50 were randomised (26 females; 24 males) from 14 sites in nine countries. Thirty‐four patients received arimoclomol and 16 received placebo (Figure 1B).

The proportion of patients completing 12 months of randomised treatment was 79.4% in the arimoclomol group and 93.8% in the placebo group (Figure 1B). In the arimoclomol group, reasons for withdrawal included safety reason (ie, adverse events; n = 3), withdrawal of consent (n = 1), fast disease progression (early escape clause; n = 2), and death from NPC progression (n = 1). In the placebo group, one patient withdrew after 1 day owing to worsening of epilepsy (considered part of disease progression; Figure 1B).

Baseline disease characteristics and demographics of patients are detailed in Table 1. All patients had a diagnosis of NPC with mutations in both NPC1 alleles. Most patients (n = 39/50) were receiving miglustat as part of routine clinical care. Baseline mean 5‐domain NPCCSS scores, 17‐domain NPCCSS scores (excluding hearing domains), and NPC‐cdb scores were higher in the arimoclomol group than in the placebo group (Table 1).

**TABLE 1:** Baseline disease characteristics and demographics (full analysis set)

	Arimoclomol (n = 34)	Placebo (n = 16)	Total (N = 50)
Age (years)			
Mean (SD)	11.5 (5.4)	10.2 (4.1)	11.1 (5.0)
Median (range)	12.5 (2‐19)	10.5 (3‐16)	11 (2‐19)
Sex, n (%)			
Male	17 (50.0)	7 (43.8)	24 (48.0)
Female	17 (50.0)	9 (56.3)	26 (52.0)
Race, n (%)			
White	32 (94.1)	13 (81.3)	45 (90.0)
Asian	1 (2.9)	1 (6.3)	2 (4.0)
Native Hawaiian or other Pacific Islander	0	1 (6.3)	1 (2.0)
Other	1 (2.9)	1 (6.3)	2 (4.0)
BMI (kg/m^2^)			
Mean (SD)	18.72 (4.15)	19.46 (3.33)	18.95 (3.89)
Median (range)	17.94 (13.9‐37.7)	18.63 (13.3‐25.8)	18.43 (13.3‐37.7)
Age at diagnosis of first neurological symptom (years)			
Mean (SD)	5.05 (3.43)	5.22 (3.87)	5.10 (3.54)
Median (range)	4.00 (0‐14.2)	3.17 (1.0‐12.0)	4.00 (0–14.2)
Age at first neurological symptom (years), n (%)			
Prenatal/perinatal (<3 months)	1 (2.9)	0	1 (2.0)
Early‐infantile (3 months to <2 years)	5 (14.7)	3 (18.8)	8 (16.0)
Late‐infantile (2 to <6 years)	17 (50.0)	7 (43.8)	24 (48.0)
Juvenile (6 to 15 years)	11 (32.4)	6 (37.5)	17 (34.0)
Adolescent/adult (>15 years)	0	0	0
Time since first NPC symptom (years)			
Mean (SD)	7.61 (4.54)	8.07 (3.75)	7.76 (4.27)
Median (range)	6.15 (0.4‐16.6)	8.10 (2.0‐14.8)	7.00 (0.4–16.6)
Time since NPC diagnosis (years)			
Mean (SD)	5.59 (4.36)	5.11 (4.14)	5.43 (4.25)
Median (range)	4.10 (0.1‐15.1)	3.00 (0.8‐14.2)	3.90 (0.1‐15.1)
Treated with miglustat			
Yes	26 (76.5)	13 (81.3)	39 (78.0)
History of seizure or epilepsy, n (%)	12 (35.3)	2 (12.5)	14 (28.0)
Baseline 5‐domain NPCCSS score			
Mean (SD)	12.1 (6.9)	9.4 (6.4)	11.2 (6.8)
Median (range)	11.5 (2.0‐24.0)	8.0 (0.0‐24.0)	10.5 (0.0‐24.0)
Baseline 5‐domain NPCCSS; individual domain scores, mean (SD)			
Ambulation score	2.5 (1.6)	2.2 (1.6)	2.4 (1.6)
Speech score	2.2(1.6)	1.6 (1.2)	2.0 (1.5)
Swallow score	1.9 (1.7)	1.3 (1.7)	1.7 (1.7)
Fine motor skills score	2.8 (1.8)	1.9 (1.8)	2.5 (1.9)
Cognition score	2.8 (1.3)	2.5 (1.5)	2.7 (1.3)
Baseline full‐scale NPCCSS score, except hearing domains			
Mean (SD)	21.2 (11.5)	17.2 (11.3)	19.9 (11.4)
Median (range)	22.0 (2–39)	16.0 (2‐44)	18.0 (2–44)
Baseline NPC‐cdb score			
Mean (SD)	46.5 (24.0)	39.2 (28.6)	44.1 (25.6)
Median (range)	45.0 (6‐84)	30.5 (4‐101)	38.0 (4–101)

Abbreviations: BMI, body mass index; NPC, Niemann‐Pick disease type C; NPC‐cdb, NPC clinical database; NPCCSS, Niemann‐Pick disease type C Clinical Severity Scale.

Subgroup baseline characteristics are summarised in Table 2. In the subgroup of patients without miglustat treatment there was an imbalance in baseline characteristics indicating a much more aggressive disease course in patients randomised to arimoclomol vs those randomised to placebo. This is based on the observation that patients on arimoclomol were younger and had a higher 5‐domain scores (mean age 7 years, mean score 13.3) than patients randomised to placebo (mean age 15 years, mean score 8.7). In addition, the 3 patients with functional double mutation were among those randomised to arimoclomol (Table 3; described in Table S1). Hence by removing the subgroup of patients without miglustat from the full cohort, the baseline characteristics in the on miglustat subgroup became more balanced across the two treatment arms.

**TABLE 2:** Baseline disease characteristics and demographics by subgroup (full analysis set per subgroup)

	Arimoclomol	Placebo	Total
**Receiving concomitant miglustat**	**n = 26**	**n = 13**	**N = 39**
Age (years)			
Mean (SD)	12.8 (4.7)	9.1 (3.6)	11.6 (4.7)
Median (range)	14.0 (2–19)	9.0 (3–16)	11.0 (2–19)
Baseline 5‐domain NPCCSS score			
Mean (SD)	11.7 (7.1)	9.6 (7.1)	11.0 (7.1)
Median (range)	10.5 (2.0–24.0)	8.0 (0‐24.0)	10.0 (0‐24.0)
Age at first neurological symptom (years)			
Mean (SD)	5.25 (3.34)	4.04 (3.20)	4.85 (3.31)
Median (range)	4.5 (0.3‐14.2)	3.0 (1.0‐11.0)	4.0 (0.3–14.2)
Patients with double functional null mutation, n	0	0	0
**No concomitant miglustat**	**n = 8**	**n = 3**	**N = 11**
Age (years)			
Mean (SD)	7.0 (5.4)	15.0 (1.7)	9.2 (5.9)
Median (range)	5.5 (2–17)	16.0 (13‐16)	7.0 (2–17)
Baseline 5‐domain NPCCSS score			
Mean (SD)	13.3 (6.1)	8.7 (2.1)	12.0 (5.6)
Median (range)	14.0 (2.0‐20.0)	8.0 (7.0‐11.0)	11.0 (2.0–20.0)
Age at first neurological symptom (years)			
Mean (SD)	4.39 (3.87)	10.33 (1.53)	6.01 (4.32)
Median (range)	3.5 (0‐12.3)	10.0 (9.0‐12.0)	6.0 (0–12.3)
Patients with double functional null mutation, n	3	0	3
**Age ≥4 years**	**n = 30**	**n = 14**	**N = 44**
Age (years)			
Mean (SD)	12.7 (4.5)	11.2 (3.2)	12.2 (4.2)
Median (range)	13.5 (4–19)	11.0 (7‐16)	12.5 (4‐19)
Baseline 5‐domain NPCCSS score			
Mean (SD)	12.0 (6.9)	10.3 (6.4)	11.5 (6.7)
Median (range)	11.5 (2.0–24.0)	9.0 (0‐24.0)	10.5 (0‐24.0)
Age at first neurological symptom (years)			
Mean (SD)	5.57 (3.29)	5.74 (3.86)	5.62 (3.44)
Median (range)	5.00 (0.3–14.2)	4.17 (1.0‐12.0)	5.00 (0.3‐14.2)
Patients with double functional null mutation, n	0	0	0
**Age <4 years**	**n = 4**	**n = 2**	**N = 6**
Age (years)			
Mean (SD)	2.5 (0.6)	3.0 (0.0)	2.7 (0.5)
Median (range)	2.5 (2–3)	3.0 (3–3)	3.0 (2‐3)
Baseline 5‐domain NPCCSS score			
Mean (SD)	12.5 (7.9)	3.5 (0.7)	9.5 (7.7)
Median (range)	14.5 (2.0‐19.0)	3.5 (3.0‐4.0)	7.5 (2.0–19.0)
Age at first neurological symptom (years)			
Mean (SD)	1.13 (1.30)	1.58 (0.59)	1.28 (1.07)
Median (range)	0.75 (0‐3.0)	1.58 (1.2‐2.0)	0.96 (0–3.0)

Abbreviations: NPC, Niemann‐Pick disease type C; NPCCSS, Niemann‐Pick disease type C Clinical Severity Scale.

**TABLE 3:** Genotype analysis of *NPC1* mutations of enrolled patients (full analysis set)

	Arimoclomol (n = 34)	Placebo (n = 16)	Total (N = 50)
Patient genotypes by mutation type, n (%)			
Double functional null	3 (8.8)	0 (0)	3 (6.0)
Double missense	16 (47.1)	11 (68.8)	27 (54.0)
Missense/functional null	15 (44. 2)	5 (31.2)	20 (40.0)

	Arimoclomol (n = 68)	Placebo (n = 32)	Total (N = 100)
Frequency of protein mutation types, n alleles (%)			
Missense	47 (69.1)	27 (84.4)	74 (74.0)
Functional null	21 (30.9)	5 (15.6)	26 (26.0)
Frameshift	12 (17. 7)	5 (15.6)	17 (17.0)
Splicing	5 (7.4)	0 (0)	5 (5.0)
Premature stop	4 (5.9)	0 (0)	4 (4.0)

	Type	Genotype
Characteristics of double functional null mutations (arimoclomol group)		
Patient 1	Frameshift/frameshift	A1108fs/A1108fs
Patient 2	Premature stop/frameshift	L860*/Q991fs
Patient 3	Splicing/splicing	R518Q/R518Q^41^

*Note*: Genotype analyses of NPC1 were sourced from historical patient records.

### 3.2 Efficacy evaluation

#### 3.2.1 Primary outcome

**FIGURE 2:**
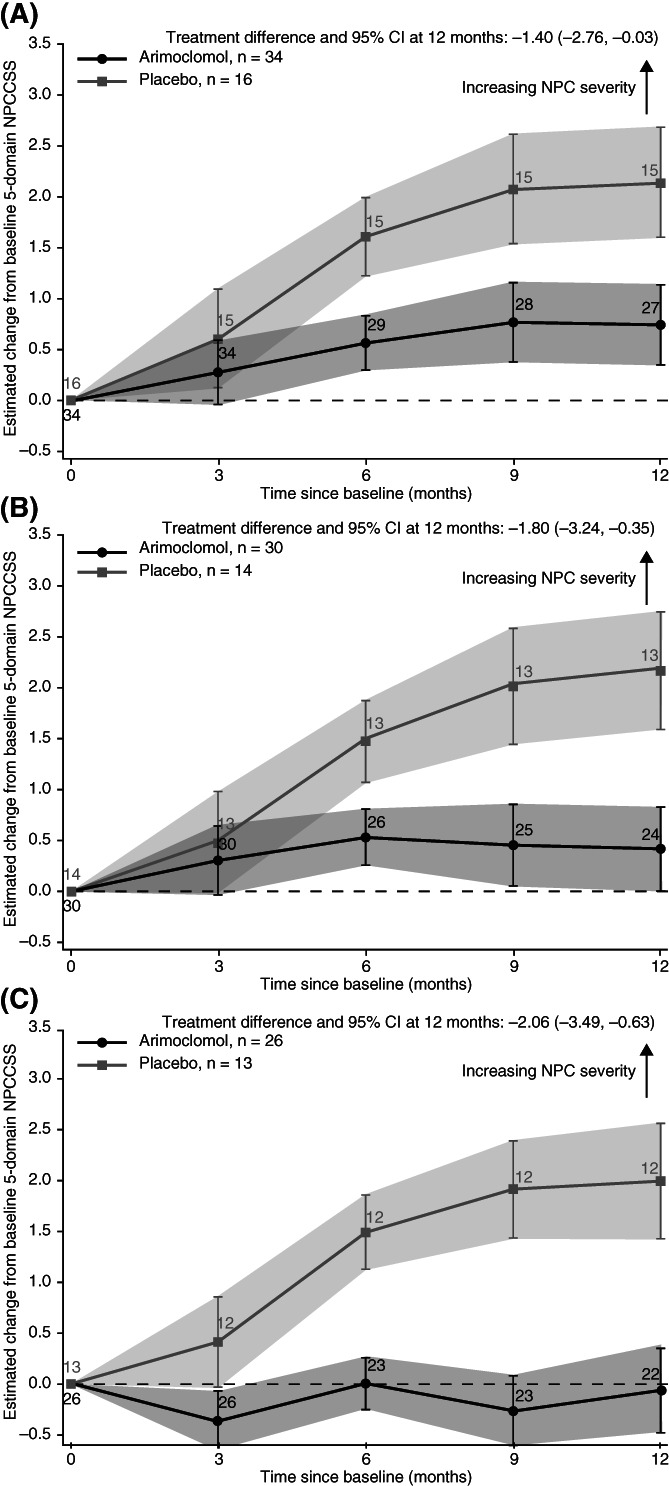
5‐domain NPCCSS score observed change from baseline at month 12: A, Overall (N = 50; full analysis set); B, in patients aged ≥4 years (n = 44); C, in patients receiving miglustat (n = 39). The solid line represents least‐squares mean estimates ± SE based on data obtained while patients were exposed to study treatment. The mixed model for repeated measures included the main effect of baseline and stratum, respectively, and interaction between treatment and visit. Change from baseline and absolute estimates correspond to the at‐baseline overall average patient. Numbers of patient are presented for each time point. CI, confidence interval; NPC, Niemann‐Pick disease type C; NPCCSS, Niemann‐Pick disease type C Clinical Severity Scale

For the primary endpoint, at month 12, mean (95% confidence interval [CI]) change on the 5‐domain NPCCSS score was 0.76 (−0.05, 1.56) for arimoclomol compared with 2.15 (1.05, 3.25) for placebo, corresponding to a significant treatment effect in favour of arimoclomol of −1.40 (95% CI: −2.76, −0.03; *P* = .046; Figure 2A) and a 65% relative reduction in annual disease progression. Patient‐level data for the change in 5‐domain NPCCSS scores are presented in Figure S1.

#### 3.2.2 Subgroup analyses of primary endpoint

In the subgroups of patients ≥4 years old (n = 44; Figure 2B) and patients concomitantly receiving miglustat (n = 39; Figure 2C), the treatment effect within each subgroup was increased (*P* < .05; Table 4).

**TABLE 4:** Change in clinical endpoints (full analysis set, except subgroup analyses)

	Arimoclomol (n = 34)	Placebo (n = 16)	Arimoclomol vs placebo: difference (95% CI)	*P* value
Change in 5‐domain NPCCSS score from baseline at 12 months				
Overall population, n (n at 12 months)	34 (27)	16 (15)		
Mean change (95% CI)	0.76 (−0.05, 1.56)	2.15 (1.05, 3.25)	−1.40 (−2.76, −0.03)	0.046
Relative reduction in annual disease progression, %			65	
Subgroup analyses				
Individuals receiving miglustat, n (n at 12 months)	26 (22)	13 (12)		
Mean change (95% CI)	−0.06 (−0.90, 0.78)	2.01 (0.85, 3.16)	−2.06 (−3.49, −0.63)	0.006
Relative reduction in annual disease progression, %			103	
Individuals not receiving miglustat, n (n at 12 months)	8 (3)	3 (3)		
Mean change (95% CI)	4.2 (1.7, 6.71)	1.99 (−1.6, 5.57)	2.21 (−2. 23, 6. 66)	0.284
Relative reduction in annual disease progression, %			NA	
Individuals aged ≥4 years, n (n at 12 months)	30 (24)	14 (13)		
Mean change (95% CI)	0.40 (−0.44, 1.24)	2.20 (1.03, 3.37)	−1.80 (−3.24, −0.35)	0.016
Relative reduction in annual disease progression, %			82	
Individuals aged <4 years, n (n at 12 months)	3 (3)	2 (2)		
Mean change (95% CI)	NC	NC		
Relative reduction in annual disease progression, %			NA	
Responders on 5‐domain NPCCSS at 12 months, n (%)	17 (50.0)	6 (37.5)	12.5 (−16.6, 41.6)	0.546
Proportion worsening on 5‐domain NPCCSS at 12 months, n (%)	15 (44.1)	7 (43.8)	0.37 (−29.1, 29.8)	1.000
Time to worsening on 5‐domain NPCCSS, months (95% CI)	5.2 (2.29, 12.0)	5.5 (1.0, 6.5)	NA	0.802
Full NPCCSS (excluding hearing domains) at 12 months, n	25	15		
Mean (SD)	1.2 (2.6)	2.7 (5.4)		
Median (Min, Max)	1.0 (−6.0, 6.0)	0.0 (−7.0, 13.0)		
Change from baseline (SE)	1.56 (0.75)	2.91 (1.02)	−1.35 (−3.91, 1.22)	0.296
Responders on CGI‐I at 12 months, n (%)	20/34 (58.8)	9/16 (56.3)	2.6 (−26.8, 32.0)	1.000
NPC‐cdb score change from baseline to 12 months, LS mean (95% CI)	1.85 (−2.16, 5.86)	4.88 (−0.63, 10.39)	−3.03 (−9.90, 3.85)	0.379
SARA score change from baseline to 12 months, LS mean (95% CI)	1.06 (−0.17, 2.29)	0.78 (−0.90, 2.47)	0.28 (−1.82, 2.37)	0.790
EQ‐5D‐3L Y proxy, n (%)				
Improved at 12 months	7/27 (25.9)	6/15 (40.0)	−14.1 (−43.9, 15.7)	0.488
Worsened at 12 months	12/27 (44.4)	3/15 (20.0)	24.4 (−3.1, 52.0)	0.180
9‐HPT time (s), change from baseline to 12 months, LS mean (95% CI)				
Dominant hand	−3.29 (−15.56, 8.98)	−6.49 (−20.34, 7.37)	3.20 (−15.71, 22.12)	0.728
Non‐dominant hand	11.68 (−14.89, 38.25)	17.59 (−13.24, 48.42)	−5.91 (−47.54, 35.72)	0.770

*Note*: The mixed model for repeated measures included the main effect of baseline and stratum, respectively, and interaction between treatment and visit. Change from baseline and absolute estimates correspond to the at‐baseline overall average patient. Numbers of patients are presented for each time point. Responder analysis was conducted using the chi‐squared test and the proportion worsening analysis was conducted using Fisher's exact test. CGI‐I responders were defined as patients whose score remained stable or showed improvement at 12 months. 5‐domain NPCCSS responders were defined as patients whose score remained stable (ie, total score for the 5‐domains being the same at month 12 as at baseline) or improves at 12 months compared to baseline (if a patient's total score at month 12 was lower than at baseline, this was an improvement). The NPC‐cdb analysis of covariance model was fitted with treatment, baseline efficacy outcome, total score, and use of miglustat as covariates. Time to worsening is summarised as 25% Kaplan‐Meier estimates, and then analysed using a log‐rank test stratified by miglustat use.

Abbreviations: 9‐HPT, nine‐hole peg test; CGI‐I, Clinical Global Impression—Improvement scale; CI, confidence interval; EQ‐5D‐3L Y, 5‐dimension, 3‐level EuroQol questionnaire, youth version; IQR, interquartile range; LS, least‐squares; NA, not applicable; NC, not calculated; NPC, Niemann‐Pick disease type C; NPC‐cdb, NPC clinical database; NPCCSS, Niemann‐Pick disease type C Clinical Severity Scale; SARA, Scale for Assessment and Rating of Ataxia.

#### 3.2.3 Secondary outcomes

Based on 5‐domain NPCCSS scores, the proportion of responders (stable or improved) was 50.0% and 37.5% in the arimoclomol and placebo groups, respectively (Table 4). At 12 months, albeit directionally in favour of arimoclomol, there was no statistical difference in change from baseline in the 17‐domain NPCCSS (excluding hearing domain) or NPC‐cdb scores for the arimoclomol vs placebo groups (Table 4). There was also no significant difference in the proportion of responders for CGI‐I between treatment groups (Table 4). Of note, for the majority of patients (42/50), trial investigators completed baseline CGI‐I assessments retrospectively.

### 3.3 Biomarkers

**FIGURE 3:**
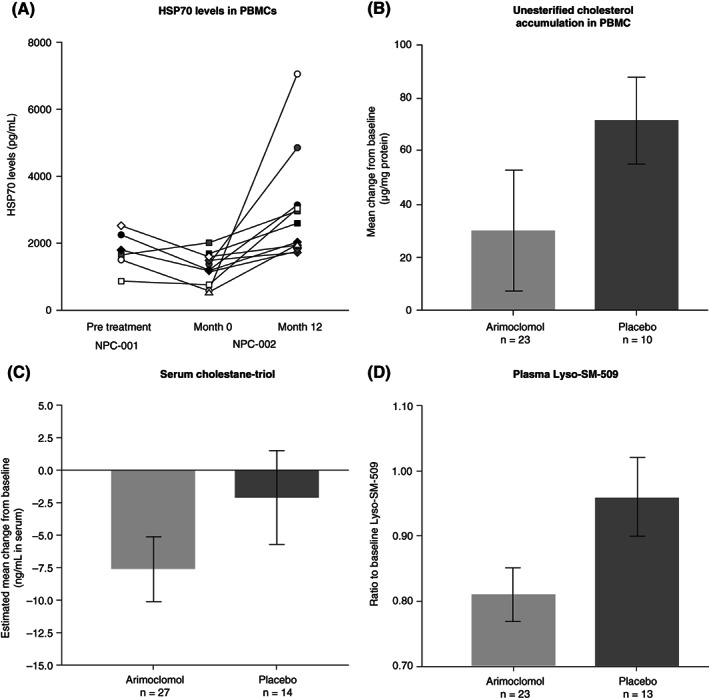
Biomarker analyses. A, Change in HSP70 in PBMCs from months 0 to 12 in arimoclomol‐treated patients (n = 11; *P* = .001); B, Change in unesterified cholesterol level at month 12 (between‐group difference: *P* = .096); C, Change in serum cholestane‐triol level at month 12 (between‐group difference: *P* = .225); D, ratio of plasma Lyso‐SM‐509 to baseline (between‐group difference: *P* = .043). Error bars show SE. Wilcoxon signed‐rank test was used to assess significance within treatment group (HSP70). For unesterified cholesterol and serum cholestane‐triols, between‐group analysis of covariance was conducted with baseline, stratum and treatment as covariates. Estimates were adjusted to reflect baseline distribution. HSP70, heat shock protein 70; NPC, Niemann‐Pick disease type C; PBMC, peripheral blood mononuclear cell

A significant increase in HSP70 level was observed in response to 12 months of treatment with arimoclomol (n = 11; mean [SD] change from baseline 1778.98 [1835.56] pg/mL; *P* = .001; Figure 3A). Because of loss of PBMC samples by the central laboratory, it was not possible to consider the placebo group (n = 4) as a control owing to the small group size. Unesterified cholesterol levels in PBMCs increased from baseline to month 12 in both placebo‐ and arimoclomol‐treated patients. However, the accumulation of unesterified cholesterol was numerically less in arimoclomol‐treated than placebo‐treated patients (mean treatment difference [SE (SE)] −44.44 [25.83] μg/mg protein; *P* = .096; Figure 3B). A non‐significant numerical decrease in serum cholestane‐triol level was observed in the arimoclomol group relative to the placebo group at 12 months (mean treatment difference [SE] −5.50 [4.46] ng/mL; Figure 3C).

All study patients had elevated levels of plasma Lyso‐SM‐509 (mean [SD (SD)] 666 [313] ng/mL) compared with healthy controls (below 10 pg/mL); baseline concentration inversely correlated with age of neurological disease onset (*P* = .002; data not shown) with a trend of increased levels with a higher 5‐domain NPCCSS score at baseline. Lyso‐SM‐509 levels showed a correlation with serum cholestane‐triol at baseline and change to Month 12 (*P* < .001; data not shown). Patients treated with arimoclomol showed a significant reduction in plasma Lyso‐SM‐509 level at Month 12 compared with placebo (*P* = .043; Figure 3D). Relative reduction in Lyso‐SM‐509 with arimoclomol correlated with plasma exposure at Month 6 and 12 (*P* = .033 and *P* = .015, respectively; Table S6).

### 3.4 Safety evaluation

In total, 88.2% (30/34) of patients in the arimoclomol group and 75.0% (12/16) in the placebo group had TEAEs (Table 5). The most common TEAE in both treatment groups was vomiting (arimoclomol: 8/34, 23.5%; placebo: 4/16, 25.0%; Table 5). Upper respiratory tract infection and decreased weight occurred more frequently with arimoclomol vs placebo, whereas nasopharyngitis, and epilepsy were reported more often with placebo vs arimoclomol (Table 5). Serious TEAEs occurred in 14.7% (5/34) of patients receiving arimoclomol compared with 31.3% (5/16) of those receiving placebo (Table 5). All serious TEAEs except those leading to discontinuation from the trial were considered related to NPC disease. One patient receiving arimoclomol died from cardiopulmonary arrest assessed as being related to NPC and not to the investigational product. Three patients in the arimoclomol group (8.8%) had four TEAEs that led to trial drug discontinuation (Table 5). These included two events of urticaria and one of angioedema (all classified as serious and as possibly related to investigational product), and one of increased blood creatinine level twice the patient's baseline value (assessed as probably related to the investigational product).

**TABLE 5:** TEAEs (safety analysis set) by Medical Dictionary for Regulatory Activities preferred term

	Arimoclomol (n = 34)	Placebo (n = 16)	Total (N = 50)
	n (%)	n (%)	n (%)
Any TEAE	30 (88.2)	12 (75.0)	42 (84.0)
Any serious TEAE	5 (14.7)	5 (31.3)	10 (20.0)
Any TEAE leading to study drug discontinuation	3 (8.8)	0 (0)	3 (6.0)
TEAEs >10% in any group			
Vomiting	8 (23.5)	4 (25.0)	12 (24.0)
Diarrhoea	7 (20.6)	3 (18.8)	10 (20.0)
Constipation	7 (20.6)	3 (18.8)	10 (20.0)
Pyrexia	6 (17.6)	3 (18.8)	9 (18.0)
Upper respiratory tract infection	6 (17.6)	1 (6.3)	7 (14.0)
Rhinitis	5 (14.7)	2 (12.5)	7 (14.0)
Weight decreased	5 (14.7)	0 (0)	5 (10.0)
Bronchitis	4 (11.8)	2 (12.5)	6 (12.0)
Nasopharyngitis	2 (5.9)	4 (25.0)	6 (12.0)
Gastroenteritis	2 (5.9)	2 (12.5)	4 (8.0)
Epilepsy^a^	1 (2.9)	2 (12.5)	3 (6.0)
Ear infection	0 (0)	2 (12.5)	2 (4.0)
Eye infection	0 (0)	2 (12.5)	2 (4.0)
Pneumonia	0 (0)	2 (12.5)	2 (4.0)
Serious TEAEs in any group			
Pneumonia	0 (0)	2 (12.5)	2 (4.0)
Urticaria	2 (5.9)	0 (0)	2 (4.0)
Angioedema	1 (2.9)	0 (0)	1 (2.0)
Aspiration bronchial	1 (2.9)	0 (0)	1 (2.0)
Cardiorespiratory arrest	1 (2.9)	0 (0)	1 (2.0)
Dysphagia	1 (2.9)	0 (0)	1 (2.0)
Epileptic encephalopathy	1 (2.9)	0 (0)	1 (2.0)
Malnutrition	1 (2.9)	0 (0)	1 (2.0)
Respiratory distress	1 (2.9)	0 (0)	1 (2.0)
Lower respiratory tract infection	0 (0)	1 (6.3)	1 (2.0)
Diarrhoea	0 (0)	1 (6.3)	1 (2.0)
Epilepsy	0 (0)	1 (6.3)	1 (2.0)
Foot deformity	0 (0)	1 (6.3)	1 (2.0)
Hypophagia	0 (0)	1 (6.3)	1 (2.0)
Laceration	0 (0)	1 (6.3)	1 (2.0)

Abbreviation: TEAE, treatment‐emergent adverse event.aThe incidence of seizure‐related TEAEs was 17.6% (n = 6/34) in the arimoclomol group and 12.5% (n = 2/16) in the placebo group.

Six patients in the arimoclomol group had an increase in serum creatinine level ≥1.5 × their baseline values; for two of these patients, levels were ≥2 × baseline values. One of the two patients continued treatment without modification of the dose, the other patient for whom the elevation was reported as a TEAE discontinued from the trial. None of the patients had any other indications of affected kidney function. For all patients, the creatinine level started to rise at the first measurement after exposure to arimoclomol and was seen to peak before the end of the trial. There were no significant changes in vital signs, electrocardiograms, or other laboratory values during the trial.

## 4 DISCUSSION

There is a significant unmet need for treatment options to slow NPC disease progression.^2^, ^4^, ^35^ However, evaluation of therapeutics for NPC is challenging given the limited number of patients and heterogeneity of NPC, and thus requires specific trial design considerations. To account for the rarity of NPC, we determined the sample size of our phase 2/3 trial through recruitment feasibility. In addition, to increase the likelihood of observing a treatment effect over 12 months, we excluded patients with adult‐onset NPC as disease progression in adults is slower than in children.^35^

In line with existing literature,^2^, ^35^ the trial population was heterogeneous with respect to disease presentation and progression rates. Compared to the placebo group, patients in the arimoclomol group were also slightly older than those in the placebo group, and the disease severity was slightly higher. The primary MMRM analysis of the 5‐domain NPCCSS included the baseline severity score as a covariate and hence controlled for the imbalance in baseline severity.

In this double‐blind, randomised, placebo‐controlled trial we observed a statistically significant (*P* = .046) treatment effect in favour of arimoclomol, corresponding to a 65% reduction in annual disease progression. The −1.40 change in the 5‐domain NPCCSS at 12 months is clinically meaningful according to the recent validation of the 5‐domain NPCCSS, which shows that a change of 1 point or greater on the scale constitutes a clinically meaningful change for caregivers/patients and physicians.^30^ Additionally, the predefined sensitivity analyses supported a treatment effect in favour of arimoclomol with a significant effect (*P* = .043) when correcting for age at first neurological symptom (Table S2). In the MMRM jackknife analysis (see Supplementary Materials), we observed that the mean treatment effect was sensitive to individual patients' data, which was especially noticeable with two placebo patients (Figure S2). However, because these two placebo patients were outliers in opposite directions, they did not markedly influence the results with respect to the central tendency.

When analysing prespecified subgroups of patients with and without miglustat as part of routine clinical care, and patients <4 years and ≥4 years, it became apparent that patients in the subgroups of patients <4 years and patients without miglustat treatment were heavily imbalanced with regards to baseline disease characteristics. In the subgroups of <4 years and without miglustat, the arimoclomol treatment group included the three patients with double functional null mutations and patients with higher baseline disease severity and younger age indicating a more aggressive disease course (Table 2). Conversely, the subgroups of patients receiving miglustat and patients ≥4 years were better balanced between treatment groups. Hence, the per‐protocol analysis of those subgroups provided an opportunity to assess whether reducing heterogeneity would result in an enhanced treatment effect. Indeed, in both subgroups, the treatment effect at month 12 with arimoclomol vs placebo was more pronounced and significant compared with the overall trial population. Compared with placebo, treatment with arimoclomol resulted in a reduction in disease progression of 82% in patients ≥4 years and disease stabilisation in patients receiving miglustat.

Although the greatest separation between arimoclomol and placebo was seen in patients receiving miglustat, the trial was not designed to assess whether there may be an additive or synergistic effect of combining the two treatments. With limited evidence in patients <4 years and the recommendation to evaluate miglustat treatment benefit on a regular basis,^36^ patients <4 years of age and patients with either no obvious progression or high progression rates were expected not to receive miglustat as part of routine clinical care. Thus, given the pronounced differences in baseline characteristics in the subgroup of patients without miglustat as part of routine clinical care, the exclusion of this subgroup seems more relevant to the assessment of the treatment effect of arimoclomol than the fact that it was assessed on top of miglustat.

To further assess the robustness of the results and explore whether other means of reducing heterogeneity would equally result in signal enhancement, we conducted additional *post hoc* analyses based on Annual Severity Increment Score (ASIS) and genotype. When applying the ASIS criteria of 0.5 to 2.0 (n = 21) as described by Cortina‐Borja et al.,^34^ we found signal enhancement with a treatment difference of −2.39 in favour of arimoclomol (*P* = .054). In addition, as expected, reducing heterogeneity by analysing only patients without double functional null mutations (n = 47) showed an enhanced signal of treatment effect of −1.61 (*P* = .020; Table S3).

Overall, the placebo group disease progression of 2.80 points on the 17‐domain NPCCSS (excluding hearing domains) over 12 months was in line with a cohort of patients of the same age, 2.92 points per year^42^; in a cohort including adults, disease progression of 1.4 to 1.9 points per year have been reported.^33^ The progression was also in line with the mean progression rate observed on the 17‐domain NPCCSS in our observational study of 2.7 points per year.^1^

Among the secondary endpoints, the NPC‐specific secondary endpoints (ie, full NPCCSS and NPC‐cbd) provided directional support of a treatment benefit of arimoclomol towards slowing disease progression. The time to worsening was based on a predefined change threshold of 2 on the overall 5‐domain NPCCSS score. Although subsequently, and as part of the 5‐domain validation, a minimal important difference threshold of 1 point or greater has been proposed.^30^ In the CGI‐I responder analysis, the proportion of patients whose disease remained stable or improved compared with baseline was similar between treatment groups. Notably, the CGI has not been validated in NPC, and assessments were introduced after initiation of the trial. Due to this, and the subsequent limitations in the completeness of data at baseline, the sensitivity to change of the CGI assessments was likely compromised.

To minimise the burden on trial patients, no cerebrospinal fluid biomarkers were taken.

Biomarker effects were determined in blood and peripheral tissue. A significant increase in HSP70 was observed in response to treatment with arimoclomol, as well as a significant reduction in plasma Lyso‐SM‐509, which correlated with arimoclomol exposure. Existing literature report that increase in Lyso‐SM‐509 in NPC correlates with NPC‐specific biomarkers and disease severity (^39^, ^40^ #43) and could be linked to lysosomal dysfunction. Lyso‐SM‐509 may be derived from sphingomyelin, a sphingolipid that accumulates in NPC and is metabolised by acid sphingomyelinase whose activity is linked directly to HSP70.^40^, ^43^ Reduction of Lyso‐SM‐509 can therefore be directly associated with the mode of action of arimoclomol through its amplification of the heat shock response. The overall impact of arimoclomol on disease‐relevant biomarkers was in line with preclinical data in NPC1 knockout mice showing central nervous system target engagement of arimoclomol, through heat shock response activation in the brain and effects on relevant disease pathology.^21^

Overall, the effect on biomarkers supports the mechanism of action of arimoclomol in NPC that involves an amplification of the heat shock response, augmentation of lysosomal function and a reduction in accumulating lipid burden.

Arimoclomol was well tolerated. The majority of TEAEs were assessed by the investigators to be related to NPC rather than the investigational product. The most common event of vomiting occurred in a similar proportion of patients in both treatment groups (arimoclomol: 8/34, 23.5%; placebo: 4/16, 25.0%). Although a higher proportion of patients in the arimoclomol vs placebo group had a history of seizures/epilepsy at baseline (35.3% vs 12.5%), a similar proportion had seizure category TEAEs (inclusive of the terms seizure, epilepsy, epileptic encephalopathy, and petit mal epilepsy) in the arimoclomol (17.6%) and placebo (12.5%) groups during the trial. Creatinine elevations were an expected pharmacodynamic effect of treatment, most likely due to inhibition of organic cation transporter 2 involved in creatinine secretion in the kidneys by arimoclomol. One patient withdrew due to increased creatinine. Serious TEAEs occurred in 5/34 (14.7%) patients in the arimoclomol group compared with 5/16 (31.3%) patients in the placebo group; 2 patients in the arimoclomol group discontinued due to serious TEAEs. One patient in the arimoclomol group died, assessed as related to NPC progression.

As with any clinical trial conducted in an ultra‐rare disorder, our study was limited by the small sample size, particularly in certain subgroups. Therefore, we acknowledge that the results are less robust than in trials with larger patient populations. The *post hoc* analyses were performed as a result of discussions with regulatory bodies.

In totality, this trial demonstrated a statistically significant and clinically meaningful treatment effect of arimoclomol in NPC supported by significant and consistent effects across several disease‐ and pharmacodynamic biomarkers. These data establish the potential of arimoclomol as an efficacious and well‐tolerated disease‐modifying treatment for NPC. The treatment effect of arimoclomol was increased in subgroups that were well balanced in terms of disease characteristics. The effect on biomarkers supports the mechanism of action of arimoclomol in NPC which involves an amplification of the heat shock response, augmentation of lysosomal function and a reduction in accumulating lipid burden. The long‐term safety and efficacy of arimoclomol is currently being further explored in the ongoing open‐label extension study.

## CONFLICT OF INTEREST

Eugen Mengel has received investigator fees and consultant honoraria from Actelion, Alexion, Orphazyme A/S, Sanofi Genzyme, and Takeda. Marc C. Patterson has received, or will receive, research support from Actelion, Amicus, Glycomine, NIH, Orphazyme A/S, and Shire/Takeda; has served as Chair of the Scientific Advisory Committee of a Registry of Niemann‐Pick disease type C, sponsored by Actelion Pharmaceuticals, Inc.; has received consultancy fees from Amicus, Genzyme, IntraBio, Novartis, Orphazyme A/S, Takeda, and Vtesse‐Sucampo‐Mallinckrodt; holds stock in IntraBio; and receives a stipend as Editor‐in Chief of the *Journal of Child Neurology* and *Child Neurology Open* (Sage), as an editor of the *Journal of Inherited Metabolic Disease* (*JIMD*) and *JIMD Reports* (Society for the Study of Inborn Errors of Metabolism), and royalties from *UpToDate* (Section Editor for Pediatric Neurology). Rosalia M. Da Riol has received travel expenses and congress fees reimbursements from Sanofi Genzyme and Takeda. Mireia Del Toro has received consulting fees and speaker honoraria, travel expenses, and congress fees from Biomarin, Sanofi Genzyme, and Takeda, and is an investigator for industrial trials (Orphazyme, Takeda, Vtesse‐Sucampo‐Mallinckrodt). Federica Deodato has received speaker honoraria from Sanofi Genzyme and Takeda, and travel reimbursement and congress fees from Actelion, Sanofi Genzyme, and Takeda. Matthias Gautschi has received consulting fees from Sanofi Genzyme, and travel expenses and congress fees from Takeda, and is an investigator for industrial trials from Horizon, Idorsia, Kaleido, Mallinckrodt, and Orphazyme A/S. Stephanie Grunewald has received consultancy funding from Hyperion, Moderna, Nutricia, Sobi, and Ultragenyx, and has participated in commercially funded research and received travel grants from Orphazyme A/S. Sabine Grønborg has received speaker honoraria from Actelion and Novo Nordisk, and travel grants from Sanofi Genzyme. Paul Harmatz has provided consulting support to and/or has received grant support from Aeglea, Alexion, Armagen, Audentis, BioMarin, Chiesi, Denali, Enzyvant, Genzyme, Homology, Inventiva, JCR, Orphazyme, Paradigm, Pfizer, PTC Therapeutics, RegenXbio, Sangamo, Shire, Sobi, and Ultragenyx. Bénédicte Héron has received consulting fees and speaker honoraria from Actelion and Takeda, travel expenses and congress fees reimbursements from Sanofi Genzyme and Takeda, and is an investigator for industrial studies and trials (Abeona Therapeutics, Chiesi Idorsia, Lysogene, Orphazyme A/S, and Vtesse‐Sucampo‐Mallinckrodt). Esther M. Maier has received fees from Sanofi. Saikat Santra has participated in commercially funded research, has received education and travel grants from Actelion and Orphazyme A/S, and has participated in commercially funded research from Vtesse‐Sucampo‐Mallinckrodt. Anna Tylki‐Szymanska has received speaker honoraria and/or travel grants from BioMarin, Chiesi, Sanofi Genzyme, and Takeda. Simon Day is a paid consultant to Orphazyme A/*S. Anne* Katrine Andreasen, Marie Aavang Geist, Nikolaj Havnsøe Torp Petersen, Linda Ingemann, Thomas Blaettler, Thomas Kirkegaard, and Christine í Dali are employees and shareholders of Orphazyme A/S. Thomas Hansen is an employee of Orphazyme A/S. Agathe Roubertie has nothing to disclose.

## AUTHOR CONTRIBUTIONS

Eugen Mengel and Christine í Dali designed the trial. All authors were involved in stages of data collection, analysis, and interpretation of the trial. Thomas Hansen conducted statistical analyses. All authors were contributors in writing the manuscript, and all read and approved the final manuscript.

## ETHICS APPROVAL AND INFORMED CONSENT

All procedures followed were in accordance with the ethical standards of the responsible committee on human experimentation (institutional and national) and with the Helsinki Declaration of 1975, as revised in 2000 (5). The trial (ClinicalTrials.gov identifier: NCT02612129) protocol and associated documentation were approved by the relevant independent ethics committees and/or institutional review boards, and written informed consent was obtained at enrolment from either the patient or their legal guardian.

## Supporting information


**Supplementary Figure S1** Patient‐level change in 5‐domain NPCCSS scores from baseline to last available data (full analysis set). NPCCSS, Niemann‐Pick disease type C Clinical Severity Scale.

**Supplementary Figure S2** Outlier analysis: Jackknifed MMRM of change from baseline to month 12 in 5‐domain NPCCSS (FAS). The primary analysis was repeated 50 times, omitting one patient at a time. Data shown as estimate ± SE. FAS, full analysis set; MMRM, mixed model for repeated measures; NPCCSS, Niemann‐Pick disease type C Clinical Severity Scale.

**Appendix** **S1**: Supporting Information

## Data Availability

The trial protocol and Statistical Analysis Plans will become publicly available. Study information will be posted on https://clinicaltrials.gov/ct2/show/NCT02612129. The data that support the findings of this trial are available from Orphazyme but restrictions apply to the availability of these data, which were used under licence for the current trial, and so are not publicly available. Data are however available from the authors upon reasonable request and with permission of Orphazyme.
