# Supplementary figures and images for: Efficacy and safety of arimoclomol in Niemann‐Pick disease type C: Results from a double‐blind, randomised, placebo‐controlled, multinational phase 2/3 trial of a novel treatment
Source: J Inherit Metab Dis. 2021 Sep 7;44(6):1463–80. doi: 10.1002/jimd.12428 (PMC9293014; doi:10.1002/jimd.12428)

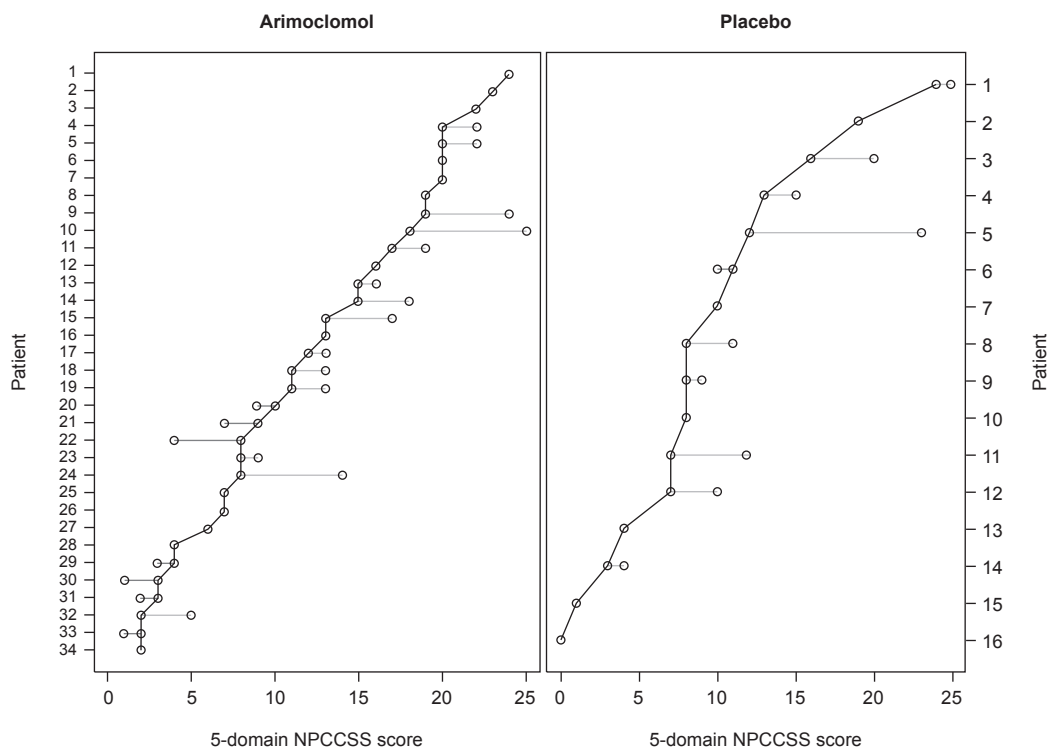

Supplement: Supplementary file 1 — Supplementary Figure S1 Patient‐level change in 5‐domain NPCCSS scores from baseline to last available data (full analysis set). NPCCSS, Niemann‐Pick disease type C Clinical Severity Scale. [file JIMD-44-1463-s001.pdf]

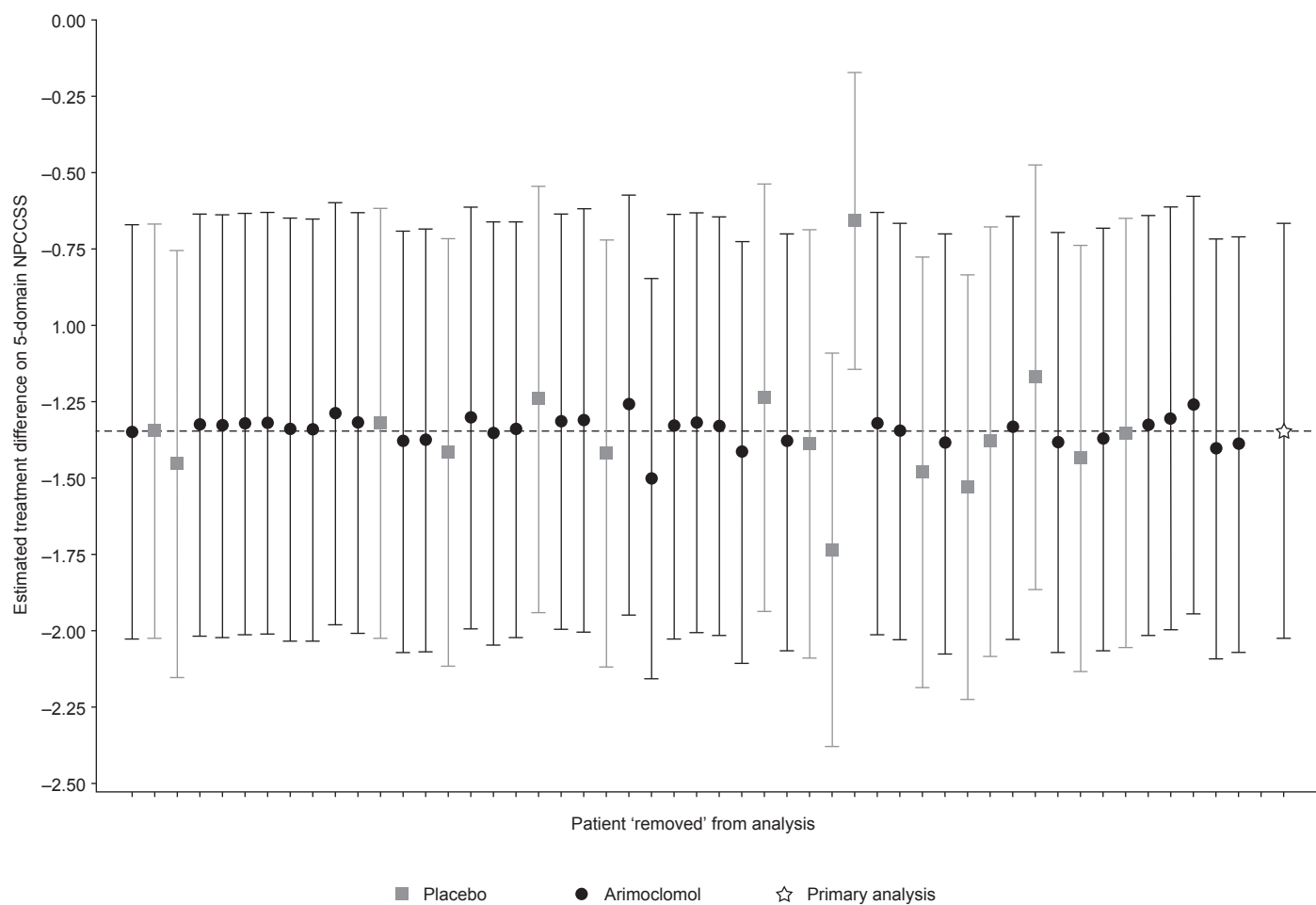

Supplement: Supplementary file 2 — Supplementary Figure S2 Outlier analysis: Jackknifed MMRM of change from baseline to month 12 in 5‐domain NPCCSS (FAS). The primary analysis was repeated 50 times, omitting one patient at a time. Data shown as estimate ± SE. FAS, full analysis set; MMRM, mixed model for repeated measures; NPCCSS, Niemann‐Pick disease type C Clinical Severity Scale. [file JIMD-44-1463-s002.pdf]
