# Supplementary material for: Efficacy and safety of arimoclomol in Niemann‐Pick disease type C: Results from a double‐blind, randomised, placebo‐controlled, multinational phase 2/3 trial of a novel treatment
Source: J Inherit Metab Dis. 2021 Sep 7;44(6):1463–80. doi: 10.1002/jimd.12428 (PMC9293014; doi:10.1002/jimd.12428)
Supplement: Supplementary file 3 — Appendix S1: Supporting Information [file JIMD-44-1463-s003.docx]

**Supplementary Materials**

**Eligibility criteria**

Eligibility criteria were chosen to allow for a sufficiently broad and representative patient population that, in order to assess a potential treatment effect, was likely to exhibit some level of progression over 12 months. With these criteria, it was sought to balance trial feasibility with the challenge to detect a treatment difference in a heterogeneous patient population with a broad age range.

Severe epilepsy with uncontrolled seizures is associated with poor prognosis leading to epileptic encephalopathy and early death. Consequently, patients who suffered from uncontrolled epilepsy were excluded from the trial due to concern of variable assessment results due to intercurrent seizures and concern of poor retention.^1^

**Randomization and masking**

Arimoclomol (or placebo) was given as an add-on therapy to the current routine clinical care. Since none of the patients were deprived from their current available therapy it was considered ethically justified to use placebo in this trial. In addition to this, the exposure to placebo was minimised as much as statistically justifiable (i.e. a randomisation scheme of 2:1 [arimoclomol:placebo] was applied).

If a dose reduction was required at visit 2 owing to increased creatinine levels, two additional patients were selected at random and dispensed new investigational product kits to mask this dose change. Only the individual requiring a dose reduction received a lower dose; the investigational product dose for the additional two patients remained unchanged.

**Procedures**

The administered dose was scaled to be equivalent to 372 mg/day for adults (with BW = 70 kg) as described in Supplementary Table 4. Based on the half-life of arimoclomol of approximately 4 hours, three times daily dosing was applied to maintain a sustained plasma exposure throughout the day.

The screening visit (visit 1) included a pharmacokinetic (PK) evaluation (in patients aged <12 years) and randomization. The arimoclomol single-dose PK evaluation was performed to confirm an acceptable drug exposure level (area under the concentration–time curve [AUC_0–‍8_] <4.6 µg*h/mL).

In the event that patients required a dose reduction, these patients were dispensed a further single dose of arimoclomol, and a new single dose PK evaluation was performed to confirm the corresponding dose. If further dose adjustments were required, based on the second single dose PK evaluation, the above procedure was repeated until the correct dose level was found.

These dose reductions took place prior to randomisation and to commencing continuous dosing of arimoclomol or placebo, as per the study randomisation.

**Dose rationale**

The dose was adjusted to a target daily exposure limit (AUC_0-24_) of 12.9 µg.h/mL in humans to ensure an adequate exposure ratio based on non-clinical toxicology studies. The final recommendations for dose stratification are listed in Supplementary Table 4.

A simple allometric scaling method using PK data from Phase I studies to relate predicted clearance (CL) (and total exposure) to body weight (BW) was selected as the most appropriate method to adjust dosage in paediatric patients with NPC because:

1. Pharmacokinetics of arimoclomol are linear (proportional) and well characterised in human adults;
2. Bioavailability of arimoclomol in human adults is high;
3. Renal clearance is expected to be a major contributor to overall elimination of arimoclomol and metabolites;
4. Maturation of renal function is considered complete at 2 years of age.

The following equation was applied to predict CL:^2^

CL in the child = adult CL×(weight of the child/70)^0.75^

**Outcome measures**

Descriptions of the outcome measures used for the primary and secondary endpoints are provided in Supplementary Table 5.

**Biomarkers**

Unesterified cholesterol

Whole blood samples were collected in K2-EDTA tubes and shipped to the central analysis site for isolation of the peripheral blood mononuclear cells (PBMCs). As part of a standardized process, pellets of 1 million cells per aliquot were generated and stored at −80°C. Lipids were extracted followed by derivatization, and unesterified cholesterol was quantified by liquid chromatography with tandem mass spectrometry (LC–MS/MS), and normalized to mg protein, as per Crosley et al.^8^

Cholestane-triol

Serum samples were collected in serum separation tubes and immediately separated by centrifugation at each clinical site. Samples were stored at −80°C and shipped to the central analysis site. Cholestane-triol was determined by LC–MS/MS.

Heath shock protein 70 (HSP70)

Whole blood samples were collected in K2-EDTA tubes and shipped to the central analysis site for isolation of the PBMCs. Standardized pellets of 1 million cells per aliquot were generated and stored at −80°C. Frozen cell pellets were homogenized in RIPA buffer with 1% protease and phosphatase inhibitor cocktail (R0278, P0044, P8340; Sigma-Aldrich, St. Louis, MO, USA), centrifuged, and extracts were diluted in assay buffer. HSP70 levels were then quantified by sandwich enzyme-linked immunosorbent assay (ELISA) (DYC1663; Research and Diagnostic Systems, Inc., Minneapolis, MN, USA).

Lysosphingomyelin-509 (Lyso-SM-509)

A relative-quantitative LC-MS/MS method was developed and validated for determination of Lyso-SM-509 in human plasma. As no reference material of Lyso-SM-509 is available, a closely related biomarker lysosphingomyelin (LysoSM) was used for preparation of calibrators and quality control (QC) samples. Due to the rarity of NPC patients and the very low concentrations of Lyso-SM-509 in healthy subjects, QC samples containing Lyso-SM-509 were prepared from dried blood spot extracts from Niemann-Pick type A/B (NP A/B) spiked into plasma. Lyso-SM-509 was assayed in human plasma by UHPLC-MS/MS analysis (positive MRM mode, m/z: 509.4 → 184.05). The deuterated analogue of LysoSM was used as an internal standard (positive MRM mode, m/z: 474.1 → 193.2). LysoSM was used for preparation of calibrators and QC (positive MRM mode, m/z: 465.3 → 184.10).

All analytical methods were validated in accordance with the FDA Bioanalytical Method Validation Guidance for Industry^9^ and met the acceptance criteria for inter-assay accuracy and precision at 15% for LC–MS/MS methods and 20% for the ELISA. No interfering peaks were detected at the retention time of the internal standard or the analytes for the chromatographic assays. No carry over was observed.

**Statistical analysis**

The 5-domain NPCCSS was analysed under two different estimands. The primary “efficacy” or “de jure” estimand assessed the effect of arimoclomol versus placebo under the assumption that patients adhered to randomized and blinded trial medication. This hypothetical assumption is relevant, as treating physicians will typically (albeit tacitly) presume that the patients will actually take the blinded trial medication. The secondary estimand, used for the sensitivity analysis, was a *de facto* “treatment policy” estimand assessing the effect of arimoclomol versus placebo in patients where there is no presumption or premise that the patients actually take the blinded trial medication; rather, the patients are simply being offered to take the blinded trial medication (i.e. the randomized patients). The crucial difference between the two estimands lie in the statistical treatment of patients (missing) data once they go off blinded trial medication. Thus, the following sensitivity analyses were conducted:

1. A multiple imputation analysis where data either missing or treated as missing in the primary analysis were simulated as if the patients had remained in the trial and remained exposed to their randomized trial medication. The analysis thus closely reflects the hypothetical nature of the estimand’s assumption on adhered to randomized trial medication.
2. Mixed model for repeated measures (MMRM) analyses akin to the primary analysis, but with the addition of the continuous main effect covariates “age at baseline” and “age at first neurological symptom”, respectively. These analyses were performed to assess the impact of these covariates, which may be unbalanced between the two treatment arms given small group sizes.

The interpretation of these two analyses differ slightly in that it is no longer just a comparison between randomized groups, but additionally between patients who share the controlled baseline characteristic.

The analysis of the “treatment policy” estimand was conducted using multiple imputation. In this analysis, however, missing data or data treated as missing was simulated regardless of which treatment the patients were randomized to, as if the patients from the time of the first missing observation either remained on placebo or switched (from arimoclomol) to placebo. The idea being that the best proxy or substitute for a patient being “off trial” after withdrawal would be the observed patterns among patients treated with placebo.

A sensitivity analysis was also performed for the “while alive and on treatment” estimand. Here, the analysis is not on the individual patients’ trajectories of 5-domain NPCCSS but, rather, simply each patient’s last post baseline assessment irrespective of administration of escape medication. The analysis was conducted with an analysis of covariance (ANCOVA) model controlling for baseline 5-domain NPCCSS and baseline miglustat use (stratification factor).

To illustrate the effects of individual patients on the overall treatment effect, a “leave-one-out jackknife” analysis was performed. The primary analysis was repeated 50 times, each time omitting a different patient one at a time. Patients exerting a large influence on the overall treatment effect will either have “unexpectedly” large or small values of change from baseline in the 5-domain NPCCSS. Patients whose behaviour follows the by-treatment mean closely will exert very little influence.

The responder analyses at 12 months used the two-tailed chi-squared test. Binary or otherwise discrete outcomes were summarized descriptively. For biomarkers, Wilcoxon signed-rank test was used to assess significance within treatment group (HSP70) between baseline and month 12. For unesterified cholesterol and serum cholestane-triols, between-group analysis of change from baseline to month 12 was conducted using an ANCOVA with baseline, stratum, and treatment as covariates. Estimates were adjusted to reflect baseline distribution. Lyso-SM-509 data were analysed using a MMRM with main effects factors for treatment, visit and the corresponding baseline value and the interaction term between visit and treatment. Estimation of plasma exposure was done using the population PK model. AUC0-8h,ss was set to zero for placebo patients. The regression analysis was performed on difference in logarithmic values at 6 or 12 months versus logarithmic values at baseline.

**Baseline Characteristics**

**SUPPLEMENTARY TABLE 1** Baseline disease characteristics and demographics by *post hoc* genetic subgroup of patients without double functional null mutations

|  | Arimoclomol | Placebo | Total |
| --- | --- | --- | --- |
| Excluding double functional null mutations | **n = 31** | **n = 16** | **N = 47** |
| Age (years), mean (SD) | 12.3 (4.8) | 10.2 (4.1) | 11.6 (4.7) |
| Baseline 5-domain NPCCSS score |  |  |  |
| Mean (SD) | 12.0 (6.8) | 9.4 (6.4) | 11.1 (6.7) |
| Median (range) | 11.0 (2.0–24.0) | 8.0 (0–24.0) | 10.0 (0–24.0) |
| Age at first neurological symptom (years), mean (SD) | 5.41 (3.35) | 5.22 (3.87) | 5.35 (3.49) |

Abbreviations: NPCCSS, Niemann–Pick disease type C Clinical Severity Scale; SD, standard deviation.

**SUPPLEMENTARY TABLE 2** Sensitivity analyses of the primary endpoint 5-domain NPCCSS (Full Analysis Set)

| Analysis method | Treatment difference  arimoclomol vs placebo (95% CI) | *p* value | Reduced progression |
| --- | --- | --- | --- |
| Sensitivity analyses of the primary estimand |  |  |  |
| Multiple imputation using data from same treatment | −1.39 (−2.83, 0.05) | 0.058 | 63% |
| MMRM – covariate: age at trial entry (years) | −1.35 (−2.73, 0.02) | 0.054 | 64% |
| MMRM – covariate: age at first neurological symptom | −1.42 (−2.78, −0.05) | 0.043 | 66% |
| Sensitivity analyses of the secondary estimand |  |  |  |
| Multiple imputation using imputation from placebo group | −1.17 (−2.97, 0.62) | 0.201 | 52% |
| While alive and on treatment estimand | −1.24 (−2.76, 0.29) | 0.110 | 55% |

Abbreviations: CI, confidence interval; FAS, full analysis set; MMRM, mixed model for repeated measures; NPCCSS, Niemann–Pick disease type C Clinical Severity Scale.

**SUPPLEMENTARY TABLE 3** Change in 5-domain NPCCSS score from baseline at 12 months in *post hoc* subgroup analyses

|  | Arimoclomol (n = 34) | Placebo (n = 16) | Arimoclomol vs placebo: difference (95% CI) | *p* value |
| --- | --- | --- | --- | --- |
| Excluding individuals with double null functional mutations, n | 31 | 16 |  |  |
| Mean change (95% CI) | 0.43 (−0.38, 1.23) | 2.03 (0.97, 3.10) | −1.61 (−2.95, −0.27) | 0.020 |
| Relative reduction in annual disease progression, % |  |  | 79 |  |
| ASIS within 0.5 and 2, n | 13 | 8 |  |  |
| Mean change (95% CI) | 0.19 (−1.26, 1.63) | 2.58 (0.65, 4.52) | −2.39 (−4.83, 0.04) | 0.054 |
| Relative reduction in annual disease progression, % |  |  | 93 |  |

The mixed model for repeated measures included the main effect of baseline and stratum, respectively, and interaction between treatment and visit. Change from baseline and absolute estimates correspond to the at-baseline overall average patient. Numbers of patients are presented for each time point. The ASIS was obtained by dividing the total 17 domain NPCCSS score by the age of the patient providing a measure of the rate of disease progression in individual patients. ASIS, annual severity increment score; CI, confidence interval; NPCCSS, Niemann–Pick disease type C Clinical Severity Scale.

SUPPLEMENTARY TABLE 4 Dosing regimen for CT-ORZY-NPC-002 trial

| **Dose (mg) per administration (t.i.d.)** | **Weight range (kg)** |
| --- | --- |
| 31 | 8–15 |
| 47 | >15–22 |
| 62 | >22–38 |
| 93 | >38–55 |
| 124 | >55 |

t.i.d, three times daily

**SUPPLEMENTARY TABLE 5 Efficacy outcome measures**

| **Efficacy Measure** | **Description** |
| --- | --- |
| NPC disease-specific clinical progression outcome measures | |
| NPC Clinical Severity Scale (NPCCSS) | - disease-specific instrument specifically designed for use in NPC to monitor disease progression and measure disease severity^3^ - total score of the 17-domain NPCCSS ranges from 0 to 61, with a higher score indicating more severe clinical impairment^3^   Note: The data for the hearing and auditory brainstem response (ABR) domains (subsequently referred to as the hearing domains) are often largely incomplete, since most patients are not able to complete the hearing test, or it would be too burdensome to perform the ABR test. Therefore, the results of the full 17-domain NPCCSS reported here exclude the hearing domains (as prespecified in the protocol). |
| NPC Clinical Database Score | - aims to reflect clinical status; an increase in score reflects a reduction in an individual’s abilities - score calculated as defined by Stampfer et al^1^ |
| Non-NPC specific outcome measures | |
| Clinical Global Impression – Improvement scale (CGI-I) | - originally developed as a research rating tool to assess psychiatric diseases^4^ - provides the clinician’s impression of improvement (or worsening) of a person’s condition at the current visit compared with baseline on a 7-point scale ranging from 1 (very much improved) to 7 (very much worse) - performed after clinical examination and patient interviews; the same investigator was instructed to perform CGI‑I assessments throughout the trial for a given patient |
| Scale for Assessment and Rating of Ataxia (SARA) | - includes 8 items reflecting neurological manifestations of cerebellar ataxia and provides a direct and simple description of motor function - total score of the eight items ranges from 0 (normal) to 40 (not able to perform any of the test items)^5^ |
| Nine-hole peg test (9-HPT) | - a direct and simple measurement of fine motor coordination, hand/eye coordination, and the ability to follow a simple direction, measured in seconds for each hand - not applicable for children under 4 years of age^6^ |
| 5-dimension 3-level EuroQol questionnaire, youth version (EQ-5D-3L Y) proxy | - child-friendly version of the EQ-5D-3L to measure health-related quality of life - questionnaire completed by the patient’s caregiver as a proxy for the individual^7^ |

**SUPPLEMENTARY TABLE 6 Regression of relative change in Lyso-SM-509 against arimoclomol exposure at Month 6 and 12**

| **Parameter Estimates** | | | | | | |
| --- | --- | --- | --- | --- | --- | --- |
| **Variable** | **Label** | **DF** | **Parameter**  **Estimate** | **Standard**  **Error** | **t Value** | **Pr > \|t\|** |
| **6m Intercept** | **Intercept** | **1** | -0.00711 | 0.05389 | -0.13 | 0.896 |
| **6m AUC_0-8h,ss_** | **AUC_0-8h,ss_** | **1** | -0.00004872 | 0.00002200 | -2.21 | 0.033 |
| **12m Intercept** | **Intercept** | **1** | -0.01898 | 0.05985 | -0.317 | 0.753 |
| **12m AUC_0-8h,ss_** | **AUC_0-8h,ss_** | **1** | -0.00006173 | 0.00002419 | -2.552 | 0.015 |

Regression of change in log-values between baseline and month 6 and 12 against estimated AUC_0-8h,ss_.

**SUPPLEMENTARY FIGURE 1** Patient-level change in 5-domain NPCCSS scores from baseline to last available data (full analysis set). NPCCSS, Niemann–Pick disease type C Clinical Severity Scale.

**SUPPLEMENTARY FIGURE 2** **Outlier analysis:** Jackknifed MMRM of change from baseline to month 12 in 5-domain NPCCSS (FAS). The primary analysis was repeated 50 times, omitting one patient at a time. Data shown as estimate ± standard error. FAS, full analysis set; MMRM, mixed model for repeated measures; NPCCSS, Niemann–Pick disease type C Clinical Severity Scale.

**References**

1. Stampfer M, Theiss S, Amraoui Y, Jiang X, Keller S, Ory D. Niemann-Pick disease type C clinical database: cognitive and coordination deficits are early disease indicators. *Orphanet J Rare Dis*. 2013;8:35. doi:10.1186/1750-1172-8-35.

2. Mahmood I. Prediction of drug clearance in children from adults: a comparison of several allometric methods. *Br J Clin Pharmacol*. 2006;61:545-57.

3. Yanjanin N, Velez J, Gropman A, et al. Linear clinical progression, independent of age of onset, in Niemann–Pick disease type C. *Am J Med Genet B Neuropsychiatr Genet*. 2010;153B:132-140.

4. Busner J, Targum SD. The clinical global impressions scale: applying a research tool in clinical practice. *Psychiatry (Edgmont)*. 2007;4:28-37.

5. Schmitz-Hubsch T, du Montcel ST, Baliko L, et al. Scale for the assessment and rating of ataxia: development of a new clinical scale. *Neurology*. 2006;66:1717-1720.

6. Poole JL, Burtner PA, Torres TA, et al. Measuring dexterity in children using the Nine-hole Peg Test. *J Hand Ther*. 2005;18:348-351.

7. EuroQol Research Foundation. EQ-5D-Y User Guide. 2020. <https://euroqol.org/publications/user-guides>. Accessed December 20, 2020.

8. Crosley LK, Duthie SJ, Polley AC, et al. Variation in protein levels obtained from human blood cells and biofluids for platelet, peripheral blood mononuclear cell, plasma, urine and saliva proteomics. *Genes Nutr*. 2009;4:95-102.

9. Food and Drug Administration. Bioanalytical Method Validation Guidance for Industry. 2018.
